# Editorial: The architecture of the human sinus node

**DOI:** 10.3389/fmed.2025.1693337

**Published:** 2025-10-01

**Authors:** Isabel M. Irurzun, Juan C. Goin, Magdalena M. Defeo

**Affiliations:** ^1^Centro de Simulación Computacional para Aplicaciones Tecnológicas-Consejo Nacional de Investigaciones Científicas y Técnicas (CSC-CONICET), Buenos Aires, Argentina; ^2^Centro de Estudios Farmacológicos y Botánicos (CEFyBO-CONICET-UBA) and II Cátedra de Farmacología, Facultad de Medicina, Universidad de Buenos Aires, Buenos Aires, Argentina; ^3^Hospital Interzonal General de Agudos “Prof. R. Rossi”, La Plata, Argentina

**Keywords:** sinoatrial node (SAN), heart rate variability (HRV), fibrosis, fractal aggregate, cardiac system visualization, Chagas

The sinoatrial node (SAN) is the primary pacemaker of the heart, where specialized cardiomyocytes spontaneously and rhythmically depolarize, causing an action potential that spreads to the atria and ventricles through a specialized conduction system. The SAN provides the basal heart rate and is also the main site from which the autonomic nervous system (ANS) regulates cardiac activity. This Research Topic addresses *The architecture of the human sinus node*, from the cellular to the systemic level.

In a healthy heart, the SAN is a cluster of cells located in the upper part of the right atrium, near the junction with the superior vena cava. High-resolution three-dimensional (3D) images obtained with micro-computed tomography showed that the SAN has a complex 3D shape with numerous radial projections (1). The study presented by Chen, Kuniewicz et al. in this Research Topic delves into the anatomy of the healthy *ex vivo* heart and includes *ex vivo* hearts with myocardial infarction. The authors constructed high-resolution 3D anatomical models combining micro-computed tomography images and computational models. In *ex vivo* hearts with myocardial infarction, the electrical impulse originates more inferiorly along the crista terminalis, aligning with what has previously been described as a “wandering pacemaker.” This can result in a benign atrial arrhythmia, common in elderly patients, with different P wave configurations on ECGs.

At the molecular level the SAN is described as a complex structure of cells with different electrophysiological profiles and intercellular coupling (2, 3). Connective tissue surrounds the SAN, electrically isolating it from the right atrium but also infiltrating it and determining its microstructure (4).

Several studies in this Research Topic highlight the role of fibrosis in the morphology of the human sinoatrial node and the surrounding right atrial muscle.

The study presented by Chen, Rams et al. showed that, with aging and obesity, extensive fibrosis and cellular hypertrophy are observed in the SAN and right atrium. Furthermore, obesity exacerbates morphological alterations, particularly hypertrophy of nodal and atrial myocytes.

In the study on Chagas disease presented by Defeo et al., an index called FN10 was proposed as a prognostic biomarker of the disease. FN10 was calculated from the HRV by using the false nearest neighbor method. It was then evaluated in a healthy population and showed a decreasing trend with age, regardless of sex, indicating mild changes in the frequency structure of HRV. Diffuse and widely dispersed cardiac fibrosis has been reported even in the early stages of chronic Chagas disease, and FN10 correlated with the level of fibrosis (5).

The study by Ricci et al. revealed that cellular heterogeneity and fibroblasts increase atrial conduction capacity. In a two-dimensional computational model, simulations showed that heterogeneity and fibroblasts, and particularly their synergistic actions, are beneficial to SAN physiology since they enlarge the parametric space in which the SAN can effectively drive the atrium.

Another study by Jorge Tasé et al. on a healthy population revealed that the complex structure of the HRV is a manifestation of the structural complexity of the SAN.

This finding implies that the complex structure of the SAN mentioned above should have self-similar characteristics. Aggregates with self-similar characteristics have scale properties that depend on both the growth rules and the spatial dimension. While their size increases with growth, their roughness (σ) initially exhibits scaling behavior, followed by a saturation regime at a crossover time (*t*_*X*_). Both *t*_*X*_ and the saturation value (σ_*sat*_) follow scaling behaviors with a characteristic length (*l*). Therefore the temporal behavior of σ is described with three scale factors, α, β, and *z*, such that *z* = α/β. A value of β = 1/3 can be proposed assuming that σ has the scaling behavior of the standard deviation of the HRV. The value of α≈1 can be estimated from measurements in mammals by establishing a scaling relationship between the body mass and *l* (6). It is interesting to note that a value of α = 1 is also obtained from the analysis of the HRV fluctuations (7), as if the scaling behavior of the HRV reflected the roughness of the SAN. Finally *z*≈3. The universal nature of the SAN architecture is defined by this set of scaling factors. Once the SAN became the dominant pacemaker, its architecture evolved into a complex structure with self-similar characteristics, leading to scale-invariant HRV.

HRV variations with age are consistent with the growth and subsequent deterioration of a self-similar structure. At early ages, apparent age-dependent scaling factors indicate the existence of a developing structure. Once complexity is fully expressed, the scaling factors stabilize at values that remain constant with age and independent of sex. At puberty, roughness reaches a saturation value. It is notable that beyond the maximum, the standard deviation of the HRV does not remain constant but decreases with sex-specific differences (8). The study by Yin et al. identified sex-specific variations in the structure and function of the sinoatrial node in the mouse heart. The authors found differences in β-adrenergic modulation between males and females, which they attributed to sex differences in the expression and activation of pacemaker currents. The authors ruled out sex differences in both channel composition and distribution. They suggested that other factors such as fibrosis, calcium channel expression and regulation, the influence of estrogen, or the innate immune system may also contribute to sex-specific differences. The reason for the decrease in the HRV standard deviation with age, beyond puberty, deserves to be further investigated. After the fourth decade of life, the scaling factors again vary with age. It is possible that a decrease in SAN roughness prevents the full expression of its scaling properties.

The process of fibrotic infiltration mentioned above can produce roughness of the SAN in the young and healthy heart. But this same mechanism, sustained over time on a limited geometry, can also lead to a decrease in roughness in old age.

The SAN is the main site from which the ANS regulates cardiac activity. It is possible that the SAN architecture has evolved such that the complexity of the ANS signal can manifest itself at the cardiac level and start cardiac cycles in fluctuating time intervals from the end of the repolarization process. Alternatively, the ANS can provide primary stimulus and the SAN can almost completely set the HRV.

The results presented in this Research Topic seem to support the idea that fibrosis plays a fundamental role in the transmission of HRV from the SAN to the atrioventricular node, and the loss of HRV may be a crucial aspect in the development of cardiac disorders. The introduction of HRV into cardiac pacing has been proposed. We emphasize the need to consider the self-similar structure of the signal. Manipulation of the SAN architecture has been proposed for therapeutic purposes, and there are many other complex physiological signals that are interpreted to originate from the ANS and manifest in other organs through neuromuscular junctions whose architecture has not been fully elucidated.

Finally, we highlight the importance of studying complex cardiac anatomy in 3D. Potyagaylo et al. proposed an approach that combines micro-computed tomography data with noninvasive CineECG images in a Mixed Reality environment. These technologies offer exciting new tools for medical training and may lead to improvements in disease diagnosis and the development of new treatments or medical devices.
